# Combination of RGB and Multispectral Imagery for Discrimination of Cabernet Sauvignon Grapevine Elements

**DOI:** 10.3390/s130607838

**Published:** 2013-06-19

**Authors:** Roemi Fernández, Héctor Montes, Carlota Salinas, Javier Sarria, Manuel Armada

**Affiliations:** 1 Centre for Automation and Robotics, CSIC-UPM, Ctra. Campo Real, Km. 0.200, La Poveda, Arganda del Rey, Madrid 28500, Spain; E-Mails: hector.montes@car.upm-csic.es (H.M.); carlota.salinas@car.upm-csic.es (C.S.); javier.sarria@car.upm-csic.es (J.S.); manuel.armada@car.upm-csic.es (M.A.); 2 Faculty of Electrical Engineering, Technological University of Panama, Panama City 0819, Panama

**Keywords:** multispectral imagery, precision viticulture, Cabernet Sauvignon, optical filters, image processing, classification, K-means

## Abstract

This paper proposes a sequential masking algorithm based on the K-means method that combines RGB and multispectral imagery for discrimination of Cabernet Sauvignon grapevine elements in unstructured natural environments, without placing any screen behind the canopy and without any previous preparation of the vineyard. In this way, image pixels are classified into five clusters corresponding to leaves, stems, branches, fruit and background. A custom-made sensory rig that integrates a CCD camera and a servo-controlled filter wheel has been specially designed and manufactured for the acquisition of images during the experimental stage. The proposed algorithm is extremely simple, efficient, and provides a satisfactory rate of classification success. All these features turn out the proposed algorithm into an appropriate candidate to be employed in numerous tasks of the precision viticulture, such as yield estimation, water and nutrients needs estimation, spraying and harvesting.

## 1. Introduction

Precision viticulture is a concept that is beginning to have an impact on the wine-growing sector of numerous countries such as Australia, Argentina, Chile, South Africa, USA, Spain, France and Portugal [1]. Precision viticulture research seeks, in essence, the same main objective of precision agriculture, that is to render production more cost-effective, maximizing crop yield and quality, while reducing environmental impacts [2,3]. One of the fundamental steps for the success of precision viticulture is the capture and processing of data related to the structure of the plants. From this information, grape yield maps can be extracted for the viticulturists or vineyard managers, giving them room for manoeuvre during the growing season, and opportunity of making more informed business decisions, such as planning logistics harvest and market preparation [4,5]. Furthermore, accurate determination of different elements of the plant can be utilized as input for obtaining greater efficiency in mechanized operations such as irrigation, spraying, pruning and harvesting [6–9]. On the other hand, the geometrical structure of a plant canopy determines its interaction with fluxes of energy. Canopy architecture and density are intimately related to crop productivity since the distribution of leaf and non-leaf surfaces influences sunlight interception and subsequent carbon assimilation and water loss [10]. Therefore, measurement of foliage can be very useful for estimating water and nutrients needs of grapevines [5,11].

In the last years, several studies aiming to provide automatic detection of grapevine elements for different applications, have been reported in the literature. In [12] the authors propose a method for detection of grapes in outdoor images using Zernike moments and colour information, and a support vector machine for the learning and recognition steps. Grape cluster and foliage detection algorithms are proposed in [13] for an autonomous selective vineyard sprayer. The algorithms were developed considering pesticide reduction as the main parameter while maintaining a minimum value of grape clusters detection rate. Shape and visual texture algorithms are proposed in [14] to detect grape berries. Berry detections are then counted and the eventual harvest yield is predicted. In [5] colour and local 3D shape reconstruction are utilised for identification of plant structure. A multi-class support vector machine classifier is then trained to classify 3D points into three semantic classes that are berry, branch and leaf. A system for detection and location, in natural environment, of bunches of grapes in colour images is also described in [15]. For detection, the system counts the number of pixels that are inside the limits of Red, Green and Blue components (044, 051, 064), (033, 041, 054), (055, 062, 075), and (018, 024, 036), for red grapes, and (102, 108, 089), (095, 104, 085), (076, 090, 078), and (083, 089, 038) for white grapes. These four centre values (colours) are experimentally determined (by trial and error) during the experimental phase. In [16] a tactile sensing technique is employed to haptically recognise grape stems by means of a multi-link manipulator. A supervised classifier based on the Mahalanobis distance is applied in [17] for characterising the grapevine canopy and assess leaf area and yield using RGB images. The method automatically processes set of images, and calculates the areas corresponding to seven different classes (grapes, wood, background, and four classes of leaf, of increasing leaf age). Each class is initialised by the user, who selects a set of representative pixels for every class in order to induce the clustering around them.

On the other hand, some systems that made use of the spectral differences in fruits and leaves have been successfully used in the past to identify fruits on plants [18,19]. In [18] a high detection rate of cucumber fruits is achieved by combining the images acquired by two cameras, one equipped with an 850 nm filter and the other with a filter in the 970 nm band. In this case, whereas leaves show approximately the same reflectance at 850 nm and 970 nm, the reflectance of cucumber fruits is at 850 nm significantly higher than at 970 nm. A multispectral analysis is also carried out in [19] to enhance citrus fruit detection. Principal component analysis was used to transform the multispectral images and to identify the wavelengths that could improve detection of fruit from the canopy background. The first three bands with the best performance were 650 nm, 600 nm and 700 nm. Unlike the two previous works in which only fruits were identified, the research presented in [20] proposes the utilization of a multispectral system for classification of sweet-pepper plant parts grown in greenhouses. Band-pass filters with centre wavelengths of 447 nm, 562 nm, 624 nm, 692 nm, 716 nm and a long-pass filter that blocks wavelengths lower than 900 nm were selected for the study.

This paper presents an automatic system that combines RGB and multispectral imagery for discrimination of Cabernet Sauvignon plant elements in natural environments, and without placing any screen behind the canopy. The system consists of a compact custom-made sensory rig that integrates a CCD camera and a servo-controlled filter wheel for the acquisition of images and a sequential masking algorithm based on the K-means method that classifies the pixels into leaves, stems, branches, fruits and background. The proposed system is intended to be used in an autonomous robotic system, without previous preparation of the vineyard. The rest of the paper is organised as follows: Section 2 describes the sensory rig that has been designed and manufactured for the acquisition of images, as well as the sequential masking algorithm proposed for the classification of the Cabernet Sauvignon plant elements. Section 3 presents the results obtained from the experimental tests, and in Section 4 results of this work are discussed. Finally, major conclusions and lines of future extensions are summarised in Section 5.

## 2. Materials and Methods

### 2.1. Sensory Rig

Commercial digital colour cameras usually include an interlaced set of red, green, and blue filters over its pixels, known as the Bayer pattern. These three filters, which makes the times of the three colour-sensitive cones in the human eyes, enable that an image can be restored realistically on many devices [21–23]. However, RGB imaging also suffers from some drawbacks, such as limited spectrum coverage and dependence on the environmental conditions. These drawbacks are clearly exhibited in the colorimetric phenomenon called metamerism, which is the matching of apparent colour of objects with different spectral power distribution. This indicates that there exist different spectral power distributions that sometimes get the same colorimetric representation [23–25].

**Figure 1.**
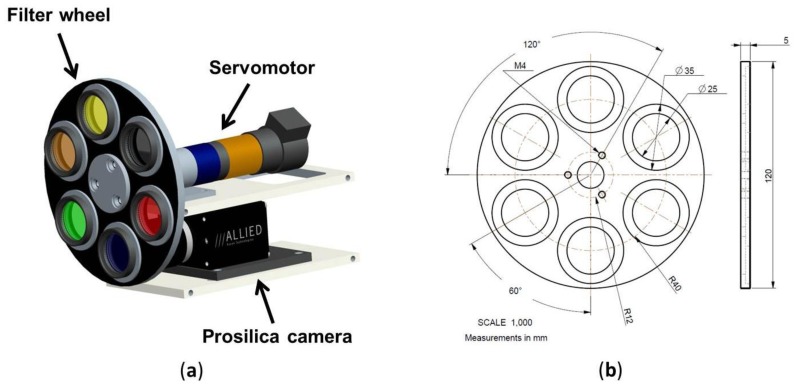
(**a**) Proposed acquisition system; (**b**) Filter wheel layout.

In order to alleviate these limitations, a multispectral system is proposed to complement the RGB image acquisition. In this way, the proposed system will enable to increase the number of spectral samples in the visible and the near-infrared range, and the performance of the classification algorithms will be improved. The system consists of a Prosilica GC2450 camera utilised in both RGB and monochrome mode, a custom-made filter wheel and a servomotor that is responsible for the accurate positioning of the filter wheel (see Figure 1). This positioning can be achieved with a maximum angular velocity of 40 rpm and a position error of 0.001°. Although the filter wheel allows interchanging up to six optical filters, in this application only three band-pass filters with centre wavelengths of 635 nm, 660 nm and 880 nm are utilised. In addition, one position will be reserved for the acquisition of RGB images.

Selection of these filters is based on several considerations. Firstly, it is well documented in the literature that all photosynthetic plants, including grapevines, are characterised by a low reflectance in red wavelengths (600 nm–700 nm) because chlorophylls (and related pigments) absorb much of the incident energy for the photosynthesis. On the other hand, in the near-infrared wavelengths (700 nm–1,300 nm) photosynthesising plants reflect large proportions of the incident sunlight [26–28]. In addition, bands between 635 nm and 680 nm have the largest contrasts between leaf and soil reflectance [29,30]. Therefore, from the reviewed literature, red and near-infrared wavelengths are suitable candidates for improving the process of discrimination among the different elements that compose a typical vineyard scene. Secondly, a hyperspectral study was conducted in laboratory conditions.

**Figure 2.**
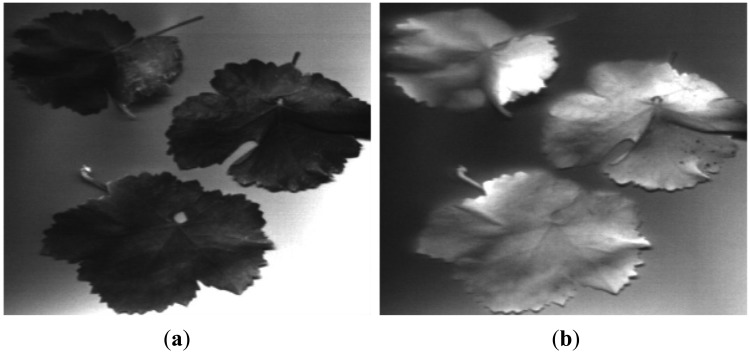
Narrow band images of grapevine leaves. (**a**) Image at 635 nm; (**b**) Image at 750 nm.

**Figure 3.**
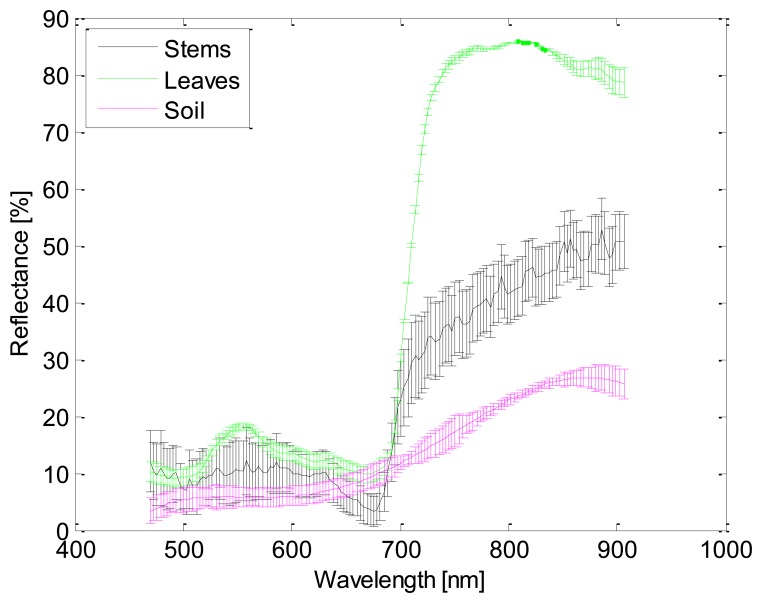
Spectral signatures.

The utilised pushbroom hyperspectral system consists of an objective lens, an ImSpector V10E spectrograph, a Pulnix TM-1327GE CCD camera and a DC-regulated 150 watt-halogen light source which provides intense, cold illumination. This system enables to record 200 spectral bands in the visible and near-infrared region between 400 nm and 1,000 nm, with 3 nm between contiguous bands. Then, several samples of the elements that will be discriminated by using the images acquired with the band-pass filters were spatially scanned, in such a way that we acquired a sequence of line images in which a complete spectrum is captured for each pixel on the line. Figure 2 shows the resulting images for leaves at 635 nm and 750 nm. With the acquired information, a spectral signature was obtained for each element of the vineyard that is intended to be discriminated by using the images acquired with the band-pass filters (see Figure 3). These elements are branches and stems, leaves and soil. Bunches of grapes are not included, since they will be discriminated by utilising the RGB images. From these signatures, the ratios of leaves-to-soil and stems-to-soil were calculated, and it was confirmed that the largest contrast of the soil with the rest of elements is attained between 630 nm and 690 nm. Near-infrared wavelengths also appear as good choices for discriminating stems from leaves. Moreover, the two wavelengths that offered the most different relative reflectances from the studied elements were around 676 nm and 886 nm. Feature reduction was also achieved by using Principal Component Analysis. The result from this procedure provides three wavelengths that can be selected for the representation of the principal components, which are: 676 nm, 758 nm, and 886 nm. Therefore, taking into account all these results and the commercial filters available in the market, a band-pass filter that has a centre wavelength of 635 nm was chosen to discriminate grapevines (leaves, stems and branches) from background (mainly soil and sky), a band-pass filter that has a centre wavelength of 880 nm was selected for discriminating the stems from the leaves, and finally, a band-pass filter that has a centre wavelength of 660 nm was picked for discriminating leaves from the remaining unclassified elements.

### 2.2. Algorithm Description

Classification techniques can be grouped into supervised and unsupervised [31–33]. Supervised classification uses *a priori* information inferred from examples, supposing to know to which class they belong, without any *a priori* definition of similarity. It is a result of an iterative procedure, which tries to find a mathematical formalism to reproduce the expert's way of assigning class memberships to patterns. The iterative process is often referred to as training or learning phase of the classifier. Besides this, parameters governing operational characteristics of the classifiers have to be identified by trial and error or by optimization procedures. Once trained, the classifier is then used to attach labels to all the image pixels according to the trained parameters [34].

Contrariwise, in unsupervised methods, the characteristics of the classes are unknown, so the classification algorithm explores the image and compute clusters that represent groups of pixels with similar spectral properties. Therefore, unsupervised classification is based on a suitable definition of similarity between patterns rather than on *a priori* knowledge of their class membership. The task of unsupervised classification can be formulated as finding groups with a minimum degree of heterogeneity, being most distant from each other. The degree of heterogeneity is defined as a distance measure, such as the Euclidean distance, the Mahalanobis distance or the adaptive determinant criterion [34].

In an unstructured outdoor scenario such a vineyard, the colour of the illumination (*i.e.*, daylight) varies with the time-of-day (sun-angle), cloud cover and other atmospheric conditions. Consequently, at different times of the day, under different weather conditions and at various positions and orientations of the targets and the sensory system, appearance of the objects could seem different [35]. This fact can hinder not only a prior identification of the features that correspond to the elements of a given class, but also, the selection of regions of interest for preparing the training set. For these reasons, the algorithm proposed in this paper is based on the K-means, one of the most popular and efficient unsupervised method [36–38]. K-means method use *K* prototypes, the centroids of clusters, to characterise the data. They are determined by minimizing the sum of squared errors:

(1) $$J_{K}=\sum_{k=1}^{K}\sum_{i\in C_{k}}{\left(x_{i}-m_{k}\right)}^{2}$$

where (*x*_1_, …, *x_n_*)= *X* is the data matrix, *m_k_* = ∑_*i*∈*C_k_*_ *x_i_*/*n_k_* is the centroid of the cluster *C_k_* and *n_k_* is the number of points in *C_k_* [39–41]. The steps of the proposed sequential masking algorithm based on the K-means method are the following.

Firstly, the K-means clustering algorithm is applied to the image acquired with the optical band-pass filter that has a centre wavelength of 635 nm, in order to partition pixels into two mutually exclusive clusters, the background and the foreground. Background includes the sky, the ground, and the weed, whereas the foreground embraces all the elements of the grapevine. Every pixel in the image is labelled in accordance with the cluster index assigned by the K-means procedure. A binary image is obtained from this preliminary result, and a morphological procedure is applied to remove small areas in the background, attaining in this way, the first mask (mask 1). This mask is applied to the RGB image and the images acquired with the optical filters whose centre wavelengths are 660 nm and 880 nm.

Next, the RGB image is transformed to L*a*b* colour space that consist of a luminosity layer ‘L*’, a chromaticity-layer ‘a*’ indicating where colour falls along the red-green axis, and a chromaticity-layer ‘b*’ indicating where the colour falls along the blue-yellow axis [42,43]. Since all the colour information exists in the ‘a*b*’ space, K-means is applied to classify the colours in ‘a*b*’ space into four clusters, which should correspond to stem and branches, leaves, fruits, and background. However, from these four clusters, only one is considered as final solution, the fruits cluster. After this procedure, a morphological operation to remove very tiny areas inside bunches of grapes is carried out, and one additional mask is obtained (mask 2). This second mask is then applied to the images acquired with the optical filters whose centre wavelengths are 880 nm and 660 nm, and K-means is utilised to classify the remaining unmasked pixels of the image of 880 nm into three groups: stems, branches and leaves. In this step, only the stem cluster (mask 3) is considered as valid and utilised for the generation of the third mask that is applied to the image acquired with the optical filter whose central wavelength is 660 nm. Then, K-means is employed by last time in order to classify the remaining unmasked pixels of the 660 nm image into three groups that are leaves, branches and all previously masked pixels. Finally, every pixel belonging to the leaves and branches clusters is labelled according to the cluster index provided by the K-means procedure, whereas the other remaining pixels are labelled according to the three mask obtained in the previous steps, corresponding to the background, fruits and stem clusters.

It also has to be mentioned that a one-time pre-processing step was required for assigning proper labels to the cluster indexes provided by the four K-means procedures that are applied throughout the proposed sequential algorithm. Therefore, assignment of labels rests on thresholds that are related to the K clusters centroid locations. Table 1 summarises the steps of the proposed algorithm.

## 3. Results

**Figure 4.**
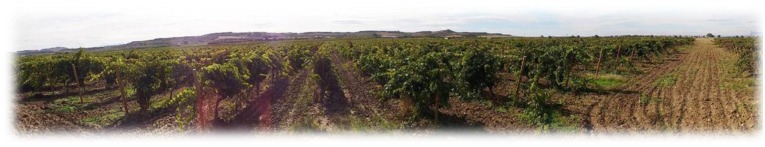
Cabernet Sauvignon vineyard.

In order to validate the proposed approach, an extensive experimental campaign was carried out. The data acquisition was conducted in October of 2012, in the commercial vineyard Dinastía Vivancos (see Figure 4), located in Haro, Spain (lat. 42°33′34.22″ N; long. 2°51′40.17″ W). Cabernet Sauvignon grapevines of this vineyard were grafted on Richter 110 and planted in 1986.

**Figure 5.**
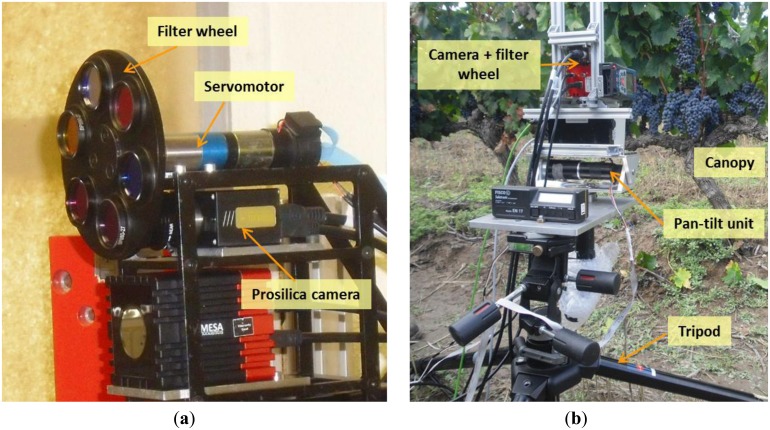
(**a**) Sensor rig close-up; (**b**) Set-up for data acquisition.

The custom-made sensory rig that integrates a CCD camera and a servo-controlled filter wheel was installed in a pan-tilt unit and mounted in a tripod set, as shown in Figure 5. This set-up was always located normal to the vineyards' canopy, at a distance of between 0.8 and 1.3 m and between 0.4 and 0.6 m aboveground. A set of images, including RGB and monochrome images with band-pass filters that have centre wavelengths of 635 nm, 660 nm and 880 nm, were captured at a resolution of 2,448 × 2,050, on both sides of the rows.

**Figure 6.**
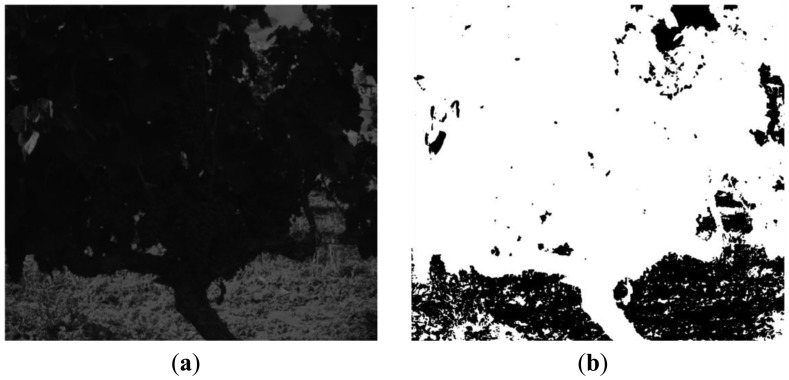
Step 1. **(a)** Image acquired with the optical filter whose centre wavelength is 635 nm; (**b**) K-means result: 2 clusters representing the background and the foreground.

**Figure 7.**
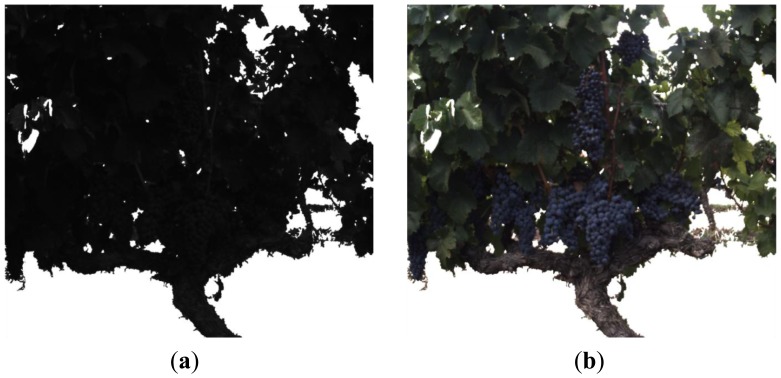
Steps 2–3. **(a)** Background mask (mask 1) obtained after morphological procedure; **(b)** RGB image with background mask.

**Figure 8.**
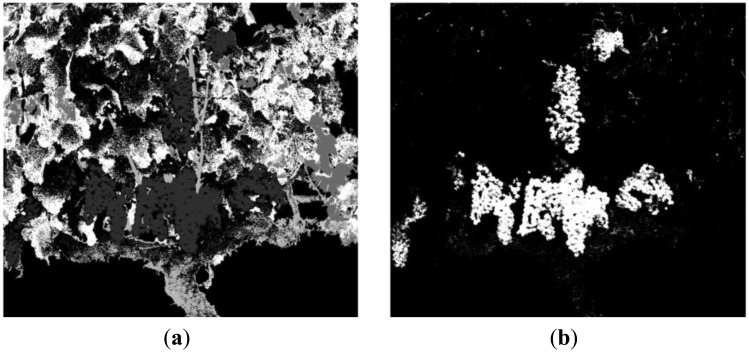
Steps 5–6. **(a)** K-means clustering applied to the ‘a*b*’ space; **(b)** Fruits mask (mask 2).

**Figure 9.**
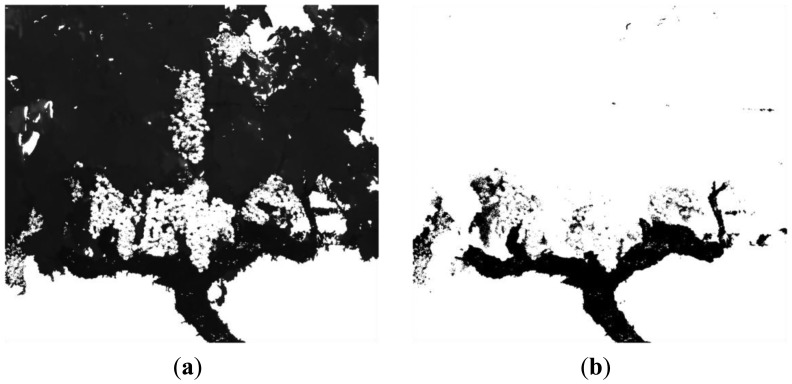
Step 8. **(a)** 880 nm image with background and fruits masked; **(b)** Stems mask (mask 3).

**Figure 10.**
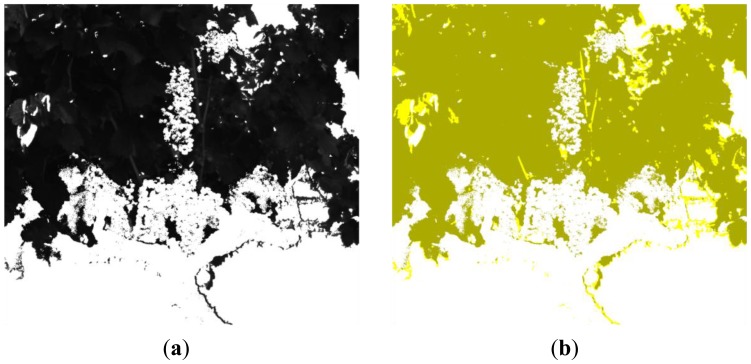
Step 9. **(a)** 660 nm image with background, fruits and stems masked; **(b)** Result of the K-means clustering.

**Figure 11.**
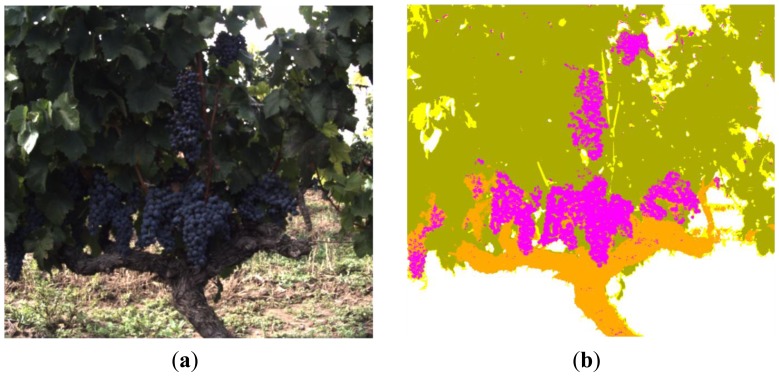
Step 10. **(a)** Original RGB image—scene 1; **(b)** Clustered image—scene 1.

**Figure 12.**
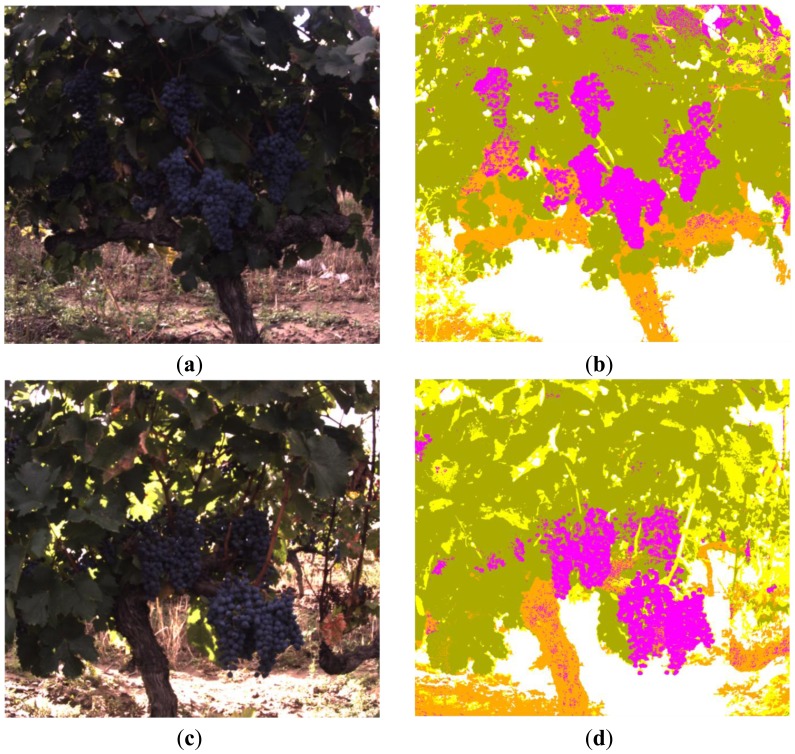
**(a)** Original RGB image—scene 2; **(b)** Clustered image—scene 2; **(c)** Original RGB image—scene 3; **(d)** Clustered image—scene 3.

**Figure 13.**
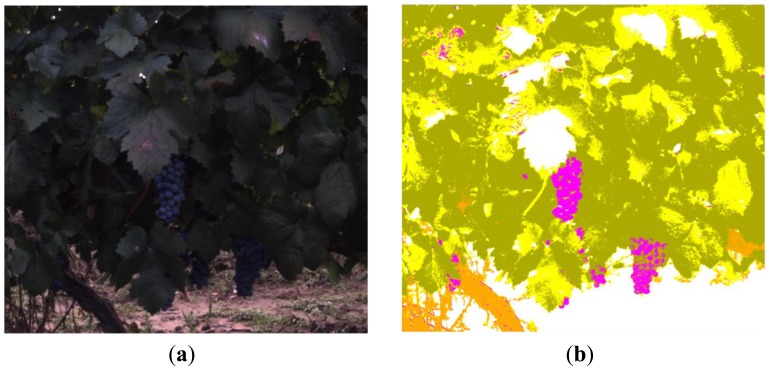

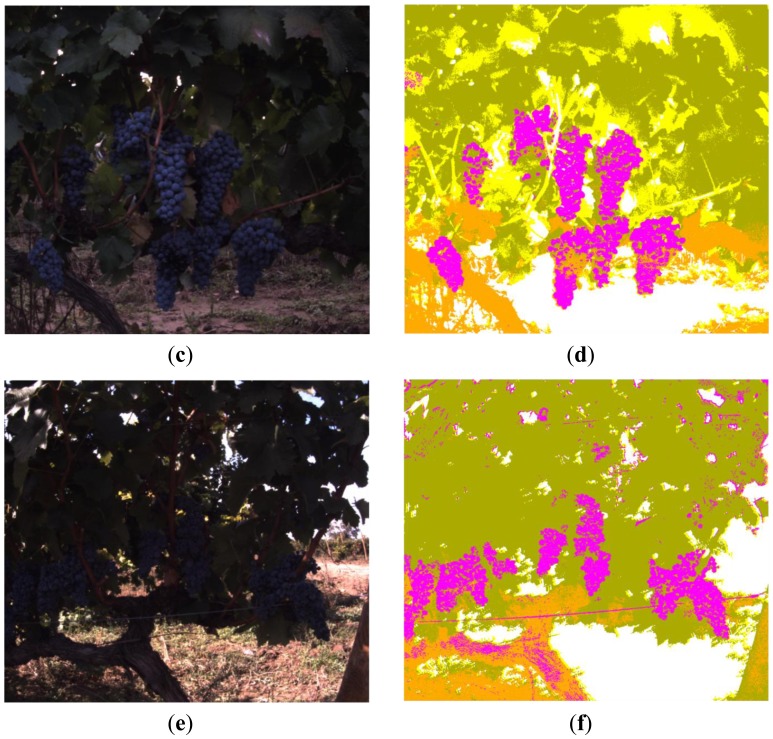
**(a)** Original RGB image—scene 4; **(b)** Clustered image—scene 4; **(c)** Original RGB image—scene 5; **(d)** Clustered image—scene 5; **(e)** Original RGB image—scene 6; **(f)** Clustered image—scene 6.

Figures 6, 7, 8, 9, 10, 11, 12 and 13 illustrate most of the intermediate results obtained from the different steps that make up the proposed algorithm. Figure 6(a) displays a scene acquired with the band-pass filter whose centre wavelength is 635 nm whereas Figure 6(b) shows the two clusters obtained from the K-means procedure, corresponding to the background and the foreground. In Figure 7 it is possible to appreciate the mask generated (mask 1) after the application of a morphological procedure to reduce small areas in the background and the RGB image with the background masked, respectively.

Figure 8(a) presents the resulting clusters after applying the K-means to the ‘a*b*’ space of the Figure 7(b). From these clusters, only the fruits cluster, shown in Figure 8(b), is utilised as final solution (mask 2). Figure 9(a) displays the remaining pixels in the image of 880 nm after applying both the background and the fruits masks. Figure 9(b) shows the stems cluster resulting from the K-means executed on the Figure 9(a) and that will be utilised as an additional mask (mask 3) in the successive steps.

Figure 10(a) displays the pixels that remain to be classified in the image acquired with the band-pass filter whose centre wavelength is 660 nm, after masking the background (mask 1), the fruits (mask 2) and the stems (mask 3). In Figure 10(b) it is possible to appreciate the three groups of pixels resulting from the K-means clustering. Green-coloured pixels belong to the leaves cluster, yellow-coloured pixels fits in the branches cluster and the rest, in white colour, are all the previously masked pixels. Finally, Figure 11(a) shows the original RGB image of the acquired scene, while the Figure 11(b) illustrates the classification result obtained with the proposed algorithm. Magenta, orange, green, yellow and white colours are utilised to visualise pixels classified as fruits, stems, leaves, branches and background, respectively.

**Figure 14.**
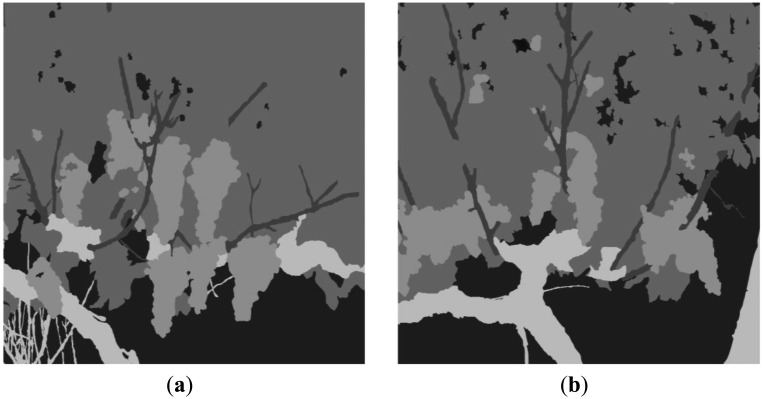
**(a)** Labelling of the image corresponding to the scene 5; **(b)** Labelling of the image corresponding to the scene 6.

Figures 12 and 13 depict classification results for five additional scenes characterised for exhibiting different lighting conditions and varied levels of occlusion. In all the presented cases the proposed algorithm demonstrated a good performance. However, to evaluate quantitatively the performance of the proposed algorithm, the original RGB images from scenes 1 to 6 were manually segmented by selecting and labelling areas corresponding to fruits, leaves, stems, branches and background. For instance, Figure 14 shows the labelling images for the scenes 5 and 6, respectively. Then, these labelled images, considered as ground truth, were compared to pixel-level with the classified images obtained from the proposed algorithm, and the matching matrix was calculated for each pair of images. With these matrixes, classification performance is assessed in terms of true-positive and false-positive detections for each class, precision for each class, total classification accuracy and total error rate [44].

The true positive rate, also called *hit rate*, *recall* and *sensitivity*, is a measure of the proportion of cases that were correctly identified, and it is defined by:

(2) $${\text{TP rate}}_{i}=\frac{\text{number of pixels of the class i correctly classified}}{\text{total number of the pixeles of the class i}}\cdot 100\%$$

The false positive rate is the proportion of pixels that were incorrectly classified as belonging to the class *i*, and it is calculated as follows:

(3) $${\text{FP rate}}_{i}=\frac{\text{number of pixels incorrectly classified}}{\text{total number of pixels of other classes different to i}}$$

Precision is a measure of the accuracy provided that a specific class has been identified. It is defined by:

(4) $$\text{Precisio}n_{i}=\frac{t_{P_{i}}}{t_{P_{i}}+f_{P_{i}}}\cdot 100\%$$

where *t_P_i__* and *f_P_i__* are the numbers of true positive and false positive predictions for the considered class *i*. Accuracy is the overall correctness of the classification algorithm and is calculated as:

(5) $$\text{Accuracy}=\frac{\text{sum of correct classifications}}{\text{total number of classifications}}\cdot 100\%$$

Finally, the error rate is given by:

(6) $$\text{Error rate}=\frac{\text{sum of the incorrect classifications}}{\text{total number of classifications}}\cdot 100\%$$

**Figure 15.**
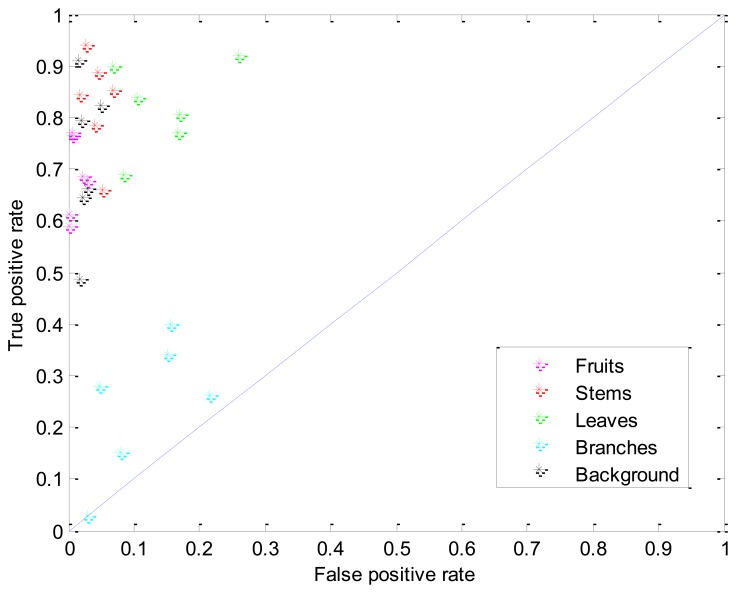
ROC graph.

Tables 2 and 3 summarise the true positive rates and the false positive rates for each class and for each scene. Higher true positive rates are attained for the Stems, Leaves and Background classes.Fruits class has a satisfactory true positive rate, reinforced for the fact of presenting a quite reduced false positive rate. On the contrary, the Branches class has a low true positive rate, and a high false positive rate in comparison with the rest of the classes. For a better visualisation of the relative tradeoffs between benefits (true positives) and costs (false positives) of the proposed algorithm, a ROC (Receiver Operating Characteristics) graph [44] is shown in Figure 15. Each pair (*TP rate*, *FP rate*) has associated a single point in the ROC space. Informally, one point in ROC space is better than another if it is to the northwest (*TP rate* is higher, *FP rate* is lower, or both) of the first [44]. Therefore, in Figure 15 it is possible to appreciate that most of the points are close to the perfect classification, represented by the point (0, 1). The Branches class is the only exception, with most of its points on the left-hand side of the ROC graph, but near the *X* axis. This performance could be understood as “conservative”: it makes positive classifications only with strong evidence so it makes few false positive errors, but it often has low true positive rates as well [44].

Table 4 gathers the precisions obtained for each class and for each scene, while Table 5 shows the accuracies and the error rates for each scene. From Table 4 it is possible to note again that higher precisions are attained for Background, Fruits and Leaves classes, whereas the lowest precision is obtained for the Branches class. Therefore, experimental results provide mean classification precisions of 89.7% for Fruits, 57.2% for Stems, 87.6% for Leaves, 5.4% for Branches and 89.2% for Background and a total mean accuracy of 75.8%.

**Figure 16.**
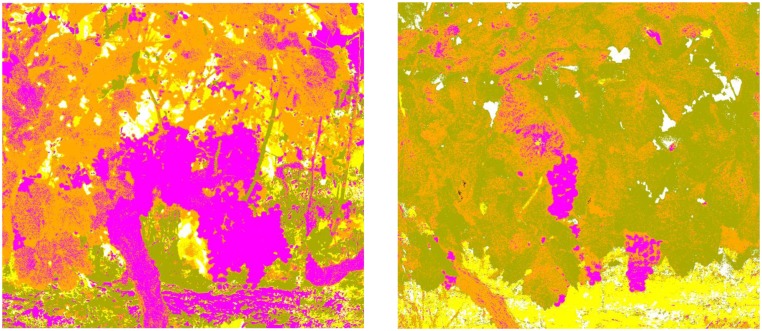
**(a)** Clustered image—scene 3; **(b)** Clustered image—scene 4.

Finally, some comparative results are presented in order to confirm that the utilisation of the combination of RGB and multispectral imagery (with properly selected band-pass filters), together with the proposed sequential masking algorithm based on the K-means method outperforms the results obtained from a simple colour based image classification using K-means clustering. For this, RGB images acquired for scenes 1 to 6 are transformed to the L*a*b* colour space, and the K-means method is applied to classify the colours in ‘a*b*’ space into five clusters, which should correspond to Stems, Branches, Leaves, Fruits and Background classes. Figure 16 depicts classification results for scenes 3 and 4, respectively. Magenta, orange, green, yellow and white colours are utilised to visualise pixels classified as Fruits, Stems, Leaves, Branches and Background, respectively. These results are then compared to pixel-level with the ground truth labelled images, and the precision, as well as the total classification accuracy and the total error rate are calculated for each class and for each scene. These quantitative results are summarised in Tables 6 and 7, respectively. Therefore, mean classification precisions obtained are 72.5% for Fruits, 9.8% for Stems, 57.8% for Leaves, 2.9% for Branches and 66.6% for background, whereas the total mean accuracy achieved is 35.1%, what confirms an enhancement of the classification results with the proposed approach.

## 4. Discussion

Gathering together the quantitative results obtained from the experimental tests presented in the previous section, it is possible to highlight that the highest hit rates of classification were attained for the Stems, Leaves and Background classes with 82.8%, 82.0% and 72.0% respectively, while the Branches class exhibited the lowest performance with a hit rate value of 24.3% and a false positive rate of 11.3%. Fruit class attained a satisfactory hit rate of 68.3%, reinforced for the fact of presenting the lowest false positive rate with a value of 1.1%. In addition, the mean classification precisions achieved from the experimental results were of 89.7% for Fruits, 57.2% for Stems, 87.6% for Leaves, 5.4% for Branches, and 89.2% for Background. All these results provide a total accuracy of 75.8%, what means that the proposed approach attains a high level of correctness in classifying the pixels of the images into the five different classes corresponding to Fruits, Leaves, Stems, Branches and Background.

A more detailed observation of experimental results brings to light that common misclassification errors are produced by atypical leaves colourations, shadows, white bright pixels wrongly assigned to the Background class and the presence of fungicide (copper sulphate). The fact that the images were acquired at a distance of between 0.8 and 1.3 m may have contributed to the low performance achieved for the Branches class, especially if we take into account the small area that makes up the branches, their characteristic cylindrical shape, and that most of these branches are affected by either shadows or occlusions. Moreover, ground truth labelling of images is done manually, and this process is not 100% free from mistakes. As branches are represented in images by a reduced number of pixels in comparison with the rest of the grapevine elements, they are more susceptible to be affected by labelling errors, what could also have contributed to shorten the final performance achieved for the Branches class.

Nevertheless, it is important to remark again that the proposed approach demonstrates a highly satisfactory performance for the classification of the grapevine elements in natural environments and without any previous preparation of the vineyard. Furthermore, the results from the utilisation of the combination of RGB and multispectral imagery (with properly selected band-pass filters), together with the proposed sequential masking algorithm based on the K-means methods surpass the results obtained from a simple colour based image classification using K-means clustering. More specifically, the proposed approach improves the mean classification precisions by 17.2 percentage points for Fruits, 47.7 percentage points for Stems, 29.8 percentage points for Leaves, 2.5 percentage points for Branches and 22.6 percentage points for Background, and the total mean accuracy in 2.2 times.

Finally, it is also important to mention some considerations regarding the lighting. Experiments were carried out along several days, with different environmental conditions (sunny and cloudy), at different hours of the days, including morning, noon and afternoon, and in both sides of the vineyard's rows. No artificial lights were utilised for illuminating the scenes during the images acquisition process. However, the orientation of the vineyard's rows with respect to the sun, and the location of the sensor rig with respect to the grapevines, which was mainly constrained by the distance between the rows, produced a uniform lighting of the scenes. Therefore, more investigations should be conducted in order to study the performance of the proposed approach in more challenging environments, and the possibly improvement of its robustness.

## 5. Conclusions and Future Work

This paper demonstrates the feasibility of identifying Cabernet Sauvignon grapevine elements in unstructured natural environments working from a combination of RGB and multispectral imagery. The solution includes a custom-made sensor rig made up of a CCD camera and a servo-controlled filter wheel, and a sequential masking algorithm based on the K-means clustering. This algorithm allows discriminating five different classes that are Leaves, Stems, Branches, Fruits and Background. Experimental results show mean classification precisions of 89.7% for Fruits, 57.2% for Stems, 87.6% for Leaves, 5.4% for Branches and 89.2% for Background and a total mean accuracy of 75.8%.

Therefore, the proposed solution enables a fast data acquisition and provides an accurate enough discrimination of grapevine elements, without any pre-treatment of the images, and without any previous preparation of the vineyard, making it suitable for many applications, such as yield estimation, leaf area estimation, spraying and harvesting.

Future work should be directed to enhance the classification performance for the Branches class. Among the steps to be investigated it could be a more extensive hyperspectral study in order to find a better combination of filters or the utilisation of an approach that combines object-based and pixel-based features. In addition, to gain understanding of what part of the algorithm is more responsible for the misclassification errors, another interesting research is to break-down the algorithm and to evaluate the performance on each step, in such a way the cause source that contributes to reduce the overall performance can be more easily determined.

## Figures and Tables

**Table 1.** Summary of the proposed algorithm.

**Step 1** **Input**: image acquired with the optical filter whose centre wavelength is 635 nm ➢ K-means clustering **Output**: 2 clusters representing the background and the foreground (binary image with 2 clusters) **Step 2** **Input**: binary image ➢ Morphological procedure to remove small areas in the background **Output**: background mask (mask 1) **Step 3** **Input**: RGB image and the images acquired with the optical filters whose centre wavelengths are 660 nm and 880 nm. ➢ Masking of the three images (using mask 1) **Output**: RGB, 660 nm and 880 nm images with the background masked **Step 4** **Input**: RGB image (with mask 1) ➢ Colour space transformation **Output**: image in the L*a*b* colour space **Step 5** **Input**: colours in ‘a*b*’ space ➢ K-means clustering **Output**: binary image with fruits cluster **Step 6** **Input**: binary image with fruits cluster ➢ Morphological procedure to remove small areas inside bunches of grapes **Output**: fruits mask (mask 2) **Step 7** **Input**: images acquired with the optical filters whose centre wavelength are 660 nm and 880 nm (both with mask 1) ➢ Masking of the image with the fruits mask (mask 2) **Output**: 660 nm and 880 nm images with background and fruits masked (mask 1 + mask 2) **Step 8** **Input**: mage acquired with the optical filter whose centre wavelength is 880 nm, with background and fruits masked (mask 1 + mask 2) ➢ K-means clustering **Output**: stems cluster – stems mask (mask 3) **Step 9** **Input**: image acquired with the optical filter whose centre wavelength is 660 nm, with background, fruits and stems masked (mask 1 + mask 2 + mask 3) ➢ K-means clustering **Output**: 3 new clusters representing branches, leaves, and all previously masked pixels **Step 10** **Input**: clusters representing branches and leaves from step 9, stems mask from step 8 (mask 3), fruits mask from step 6 (mask 2) and background mask from step 2 (mask 1) ➢ Labelling of the pixels **Output**: pixels in the image classified into five clusters that are leaves, branches, stems, fruits and background.

**Table 2.** True positive rates for each class and for each scene obtained with the proposed approach.

**Classes**	**Scene 1**	**Scene 2**	**Scene 3**	**Scene 4**	**Scene 5**	**Scene 6**
Fruits	76.5%	67.7%	77.1%	58.9%	61.1%	68.6%
Stems	94%	88.8%	85.1%	84.3%	78.5%	66%
Leaves	90%	83.7%	80.6%	68.7%	77%	92.1%
Branches	28%	15%	34%	26.1%	39.8%	2.8%
Background	91.2%	79.5%	48.6%	82.3%	66.2%	64.5%

**Table 3.** False positive rates for each class and for each scene obtained with the proposed approach.

**Classes**	**Scene 1**	**Scene 2**	**Scene 3**	**Scene 4**	**Scene 5**	**Scene 6**
Fruits	0.6%	2.9%	0.6%	0.1%	0.2%	2.1%
Stems	2.6%	4.4%	6.75%	1.6%	4.1%	5.1%
Leaves	6.9%	10.5%	16.8%	8.5%	16.8%	26%
Branches	4.7%	7.9%	15.1%	21.5%	15.4%	2.9%
Background	1.5%	1.8%	1.8%	4.9%	3%	2.2%

**Table 4.** Precisions for each class and for each scene obtained with the proposed approach.

**Classes**	**Scene 1**	**Scene 2**	**Scene 3**	**Scene 4**	**Scene 5**	**Scene 6**
Fruits	95%	77.1%	93.4%	93.6%	98%	80.9%
Stems	72.6	58.3%	42.5%	61.1%	57.2%	51.6%
Leaves	93.8%	89.5%	80.9%	96.1%	86%	79.2%
Branches	7.1%	4.5%	6.8%	1%	8.7%	4.5%
Background	95.7%	94%	93.2%	78.4%	83.4%	90.6%

**Table 5.** Accuracies and error rates for each scene obtained with the proposed approach.

	**Scene 1**	**Scene 2**	**Scene 3**	**Scene 4**	**Scene 5**	**Scene 6**
Accuracy	88.1%	79.2%	68.3%	70.9%	71.5%	76.5%
Error rate	11.9%	20.8%	31.7%	29.1%	28.5%	23.5%

**Table 6.** Precisions for each class and for each scene.

**Classes**	**Scene 1**	**Scene 2**	**Scene 3**	**Scene 4**	**Scene 5**	**Scene 6**
Fruits	89.3%	76.1%	43.9%	54.7%	97.8%	72.9%
Stems	12.1%	11.5%	2.4%	5.1%	18.6%	9.1%
Leaves	77.9%	65.1%	5.0%	93.8%	84.7%	19.0%
Branches	1.9%	1%	1.5%	1.2%	10.8%	1.21%
Background	79.1%	85.4%	76.8%	56.8%	23.7%	78%

**Table 7.** Accuracies and error rates for each scene.

	**Scene 1**	**Scene 2**	**Scene 3**	**Scene 4**	**Scene 5**	**Scene 6**
Accuracy	37.4%	28.4%	15.4%	52.1%	58.0%	19.3%
Error rate	62.6%	71.6%	84.6%	47.8%	42.0%	80.7%
